# Dr. Robert Watts Eastman

**Published:** 1913-07

**Authors:** 


Dr. Robert Watts Eastman, Buffalo 1879, born in Oswego in 1853, died at his home in New York City, May 5.

